# Precocious Puberty as a Unique Presentation of Hepatoblastoma in a Pediatric Patient: A Case Report

**DOI:** 10.7759/cureus.75114

**Published:** 2024-12-04

**Authors:** Rachel Siretskiy, Monique Motta, Gabriela Aitken, Chelsea Spector, Claudia Rojas, Jill Whitehouse

**Affiliations:** 1 Surgery, Florida International University, Herbert Wertheim College of Medicine, Miami, USA; 2 Pediatrics, Joe DiMaggio Children's Hospital, Hollywood, USA; 3 Surgery, Memorial Healthcare System, Miami, USA; 4 Anatomic and Clinical Pathology, Pediatric Pathology, Memorial Healthcare System, Hollywood, USA; 5 Pediatric Surgery, Joe DiMaggio Children's Hospital, Hollywood, USA

**Keywords:** hepatoblastoma, paraneoplastic, pediatric, precocious puberty, surgery

## Abstract

Hepatoblastoma is a rare pediatric cancer. Hepatoblastoma typically presents asymptomatically with an enlarging abdominal mass but can be associated with paraneoplastic secretion of beta-gonadotropin leading it to present like peripheral precocious puberty.

This case highlights a rare initial presentation of hepatoblastoma as precocious puberty in a two-year-old patient who presented with persistent abdominal and genital pain. The patient was treated with right hepatic lobectomy and subsequently two cycles of cisplatin and was considered to be in remission without concern for recurrent disease.

Considering that there have been more reports of this pathology in recent years, it is crucial to discuss the presentation, diagnosis, and treatment of this pathology. This case emphasizes the importance of maintaining hepatoblastoma as a differential diagnosis when evaluating precocious puberty and abdominal pain to avoid delays in diagnosis and treatment. This article also highlights our team's approach to the workup and treatment of this pathology, which can serve as a roadmap for other clinicians.

## Introduction

Hepatoblastoma is the most common primary malignant hepatic tumor in children. Hepatoblastoma accounts for 79% of liver cancers in children under the age of 15 but only makes up one percent of all pediatric cancers [1]. Hepatoblastoma typically presents asymptomatically with an enlarging abdominal mass, elevated alpha-fetal protein (AFP), and less commonly with anorexia, weight loss, and pain [2]. Some cases have reported the production of ectopic adrenocorticotropic hormone (ACTH), parathyroid hormone-related peptide (PTHRP), and beta-human chorionic gonadotropin (hCG), presenting with a paraneoplastic presentation of precocious puberty, which is speculated to be related to the ectopic beta-hCG secretion [3-9].

Precocious puberty is defined as early signs of sexual development in children. The age of onset of precocious puberty is defined as eight in female patients and nine in male patients. In male patients, signs and symptoms of precocious puberty include penile and testis enlargement, pubic or axillary hair growth, deepening of voice, and development of body odor [3]. In female patients, this precocious puberty would present as early breast development, axillary hair growth, menstruation, and rapid height growth [10]. The exact mechanism by which hepatoblastoma causes precocious puberty is still unknown. However, reports of this rare presentation have been increasing over the last three decades as indicated by the increased number of reported cases. However, delays in diagnosis as with our patient are still prevalent, which indicates a need to highlight this presentation for diagnostic application.

## Case presentation

We present a two-year-old male patient with persistent abdominal and genital pain for three months. His weight was 25.4 kg, and his height was 105 cm, both of which are above the 99th percentile for his age group. A physical exam revealed Tanner stage three pubic hair, a mature-appearing phallus, a large rugated scrotal sac, and deepened voice. Laboratory studies revealed normal liver function, ACTH, and cortisol levels. Testosterone was noted as 666 ng/dL (normal <9 ng/dL), dehydroepiandrosterone (DHEA) sulfate was 16 mcg/dL (normal <15), androstenedione was 50 ng/dL (normal <22), 17-hydroxyprogesterone was 256 ng/dL (normal <134), and beta-hCG was 72 mIU/mL (normal <50).

**Figure 1:**
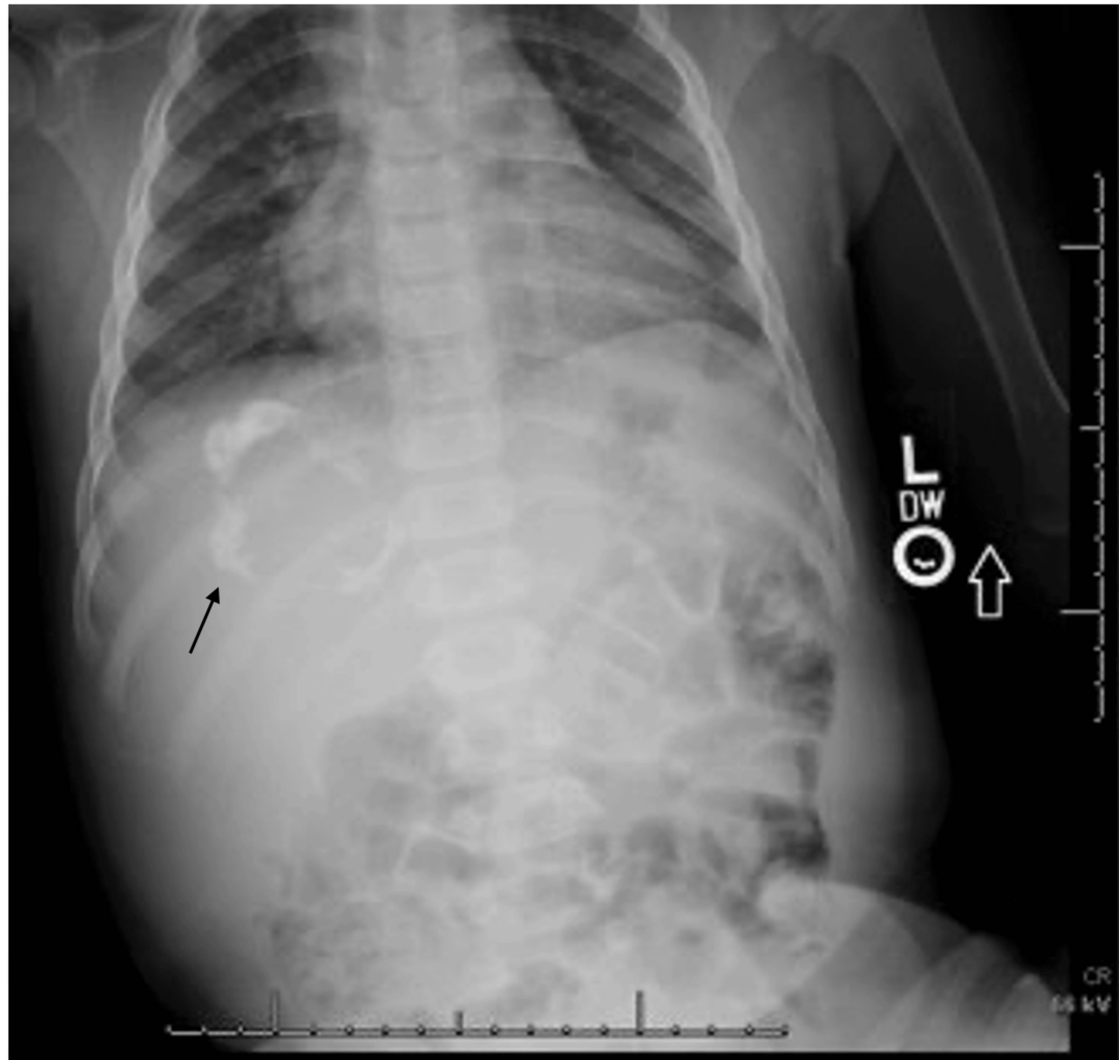
X-ray chest and abdomen: calcification in the right upper quadrant, concerning for calcified mass.

**Figure 2:**
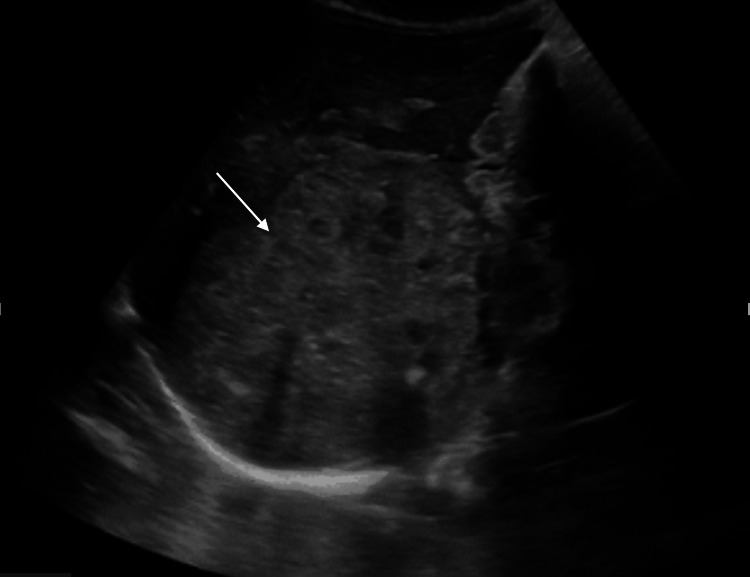
Abdominal right upper quadrant ultrasound showing soft tissue mass measuring 8.1 x 7.9 x 7.2 cm.

**Figure 3:**
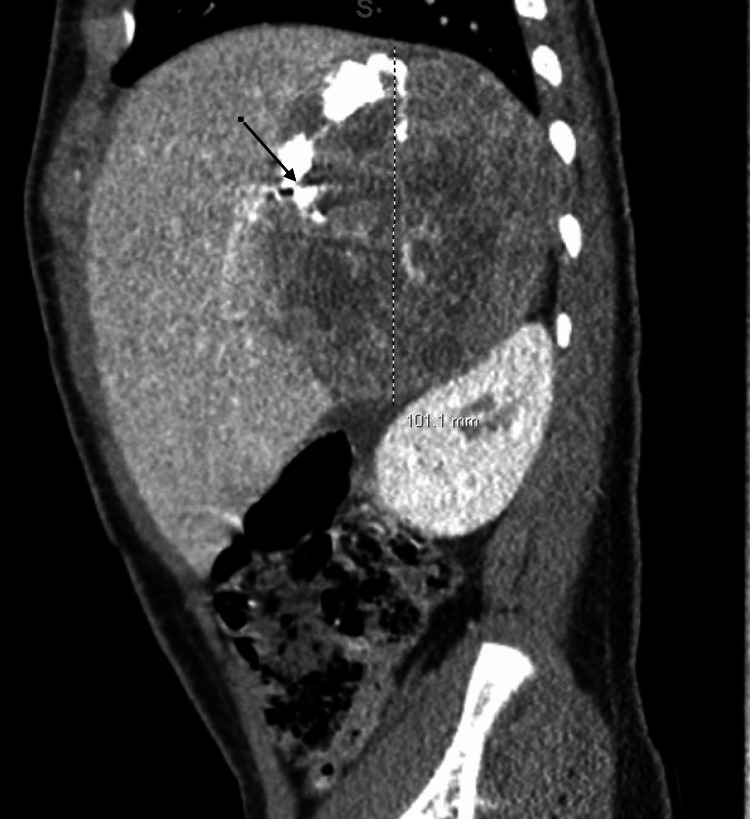
CT abdomen/pelvis with PO/IV contrast: large heterogeneously enhancing right suprarenal mass measuring approximately 10.1 x 8.7 x 8.4 cm.

An abdominal X-ray revealed course calcifications within the right upper quadrant (Figure 1). Abdominal ultrasound revealed a right upper quadrant soft tissue mass measuring 8.1 x 7.9 x 7.2 cm, which contained hyperechoic shadowing calcifications and internal vascularity (Figure 2). CT of the chest, abdomen, and pelvis revealed a large heterogeneously enhancing right suprarenal mass measuring approximately 10.1 x 8.7 x 8.4 cm with calcifications and surrounding the intrahepatic inferior vena cava (IVC) and abutting the right hepatic vein (Figure 3).

**Figure 4:**
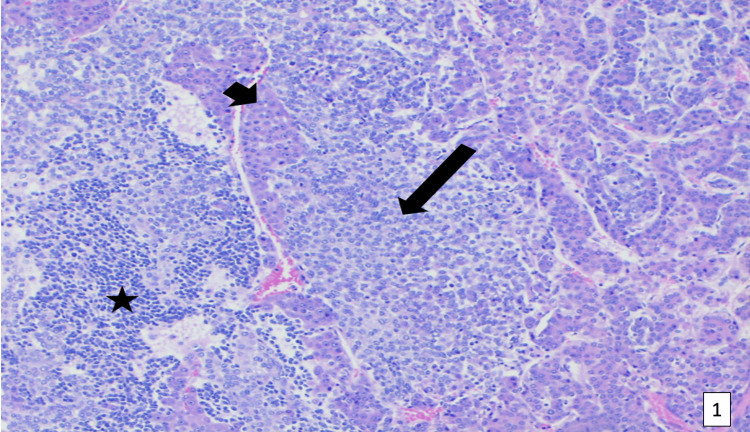
Microphotograph of epithelial-type hepatoblastoma with three subtypes: fetal subtype (short arrow) with closely packed cells that have a central nucleus and eosinophilic cytoplasm; embryonal subtype (long arrow) cells with increased nuclear-cytoplasmic ratio, scant cytoplasm with indistinct borders, and large angulated to oval nucleoli with prominent nucleoli; and small-cell undifferentiated cells, round to oval-shaped with scant cytoplasm.

**Figure 5:**
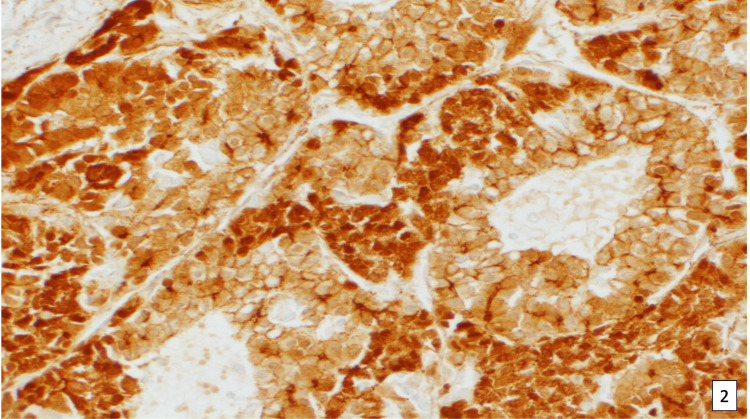
Microphotograph of fetal and embryonal subtype hepatoblastoma showing beta-catenin immunohistochemical stain.

**Figure 6:**
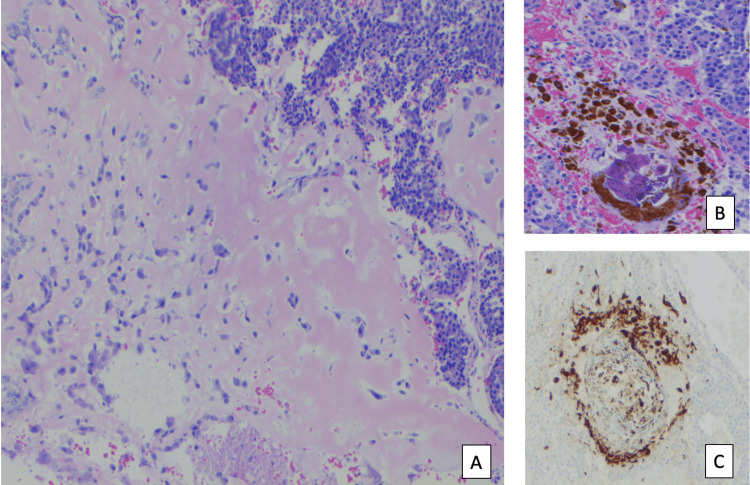
Microphotograph showing the mesenchymal component of the tumor: (A) microphotograph showing the mesenchymal component with osteoid and osteoid-like tissue; (B) microphotograph of calcification and melanin-producing cells; (C) pan melanocyte immunostaining of the tumor.

A definitive diagnosis of hepatoblastoma was made via ultrasound-guided biopsy. The tumor was staged as a Pretext 1. It encompassed the right posterior section of the liver, without caudate or additional hepatic foci, that partially encased the right hepatic vein and intrahepatic IVC. There was no evidence of tumor rupture, portal vein involvement, or distant metastasis. At this time, AFP was measured as 113,601 ng/mL (normal 0.5-7.9 ng/mL). Histological evaluation demonstrated epithelial hepatoblastoma with three subtypes, which included fetal subtype, embryonal subtype, and small-cell undifferentiated cells (Figure 4). Furthermore, membranous staining highlighted the fetal and embryonal subtypes, while the nuclear was positive in the embryonal subtype in beta-catenin staining (Figure 5). Lastly, microphotographs demonstrated that the mesenchymal portion of the tumor consisted of osteoid and osteoid-like tissue and that the calcifications had melanocytes as part of their composure (Figure 6).

The tumor was determined to be resectable, so the patient underwent a right hepatic lobectomy with right retroperitoneal nodal sampling. Final pathology identified a 10.5 x 8.5 x 5.0-cm hepatoblastoma that consisted of mixed epithelial (fetal) and mesenchymal (small-cell undifferentiated pattern (5%)) types and mitotically active with teratoid features. Parenchymal margins were negative for tumor, and lymphovascular invasion was not identified. Post-operatively, the patient recovered well and was discharged home on post-operative day nine. After resection, AFP levels decreased to 8,563.0 ng/mL. The patient went on to receive two cycles of cisplatin therapy. After his first round of cisplatin, his AFP dropped to 28.7 ng/mL, and after the second round, his AFP was within normal limits at 5.5 ng/mL. Beta-hCG remained within normal limits after treatment, and a repeat CT scan showed no evidence of pulmonary metastasis or local disease recurrence. The patient was determined to be in remission, and therefore, his port was removed.

## Discussion

There have been approximately 40 reported cases of hepatoblastoma presenting as precocious puberty [4]. While the true mechanism by which hepatoblastoma leads to the presentation of precocious puberty is still unknown, there are several theories that speculate on the etiology of this relationship. The two potential hypotheses for the paraneoplastic manifestation of precocious puberty secondary to hepatoblastoma are the following: (1) beta-hCG produced by the tumor stimulates Leydig cells to produce testosterone and (2) beta-hCG’s structural similarity to luteinizing hormone and follicle-stimulating hormone leads to direct testosterone secretion in a gonadotropin-releasing hormone-independent manner [5]. It is imperative to note that all reported cases of virilizing hepatoblastoma were seen in patients who presented with elevated beta-hCG levels, highlighting the central role that it has in the development of this presentation [6].

Early studies suggested that virilizing hepatoblastoma carried worse outcomes compared to non-virilizing, but more recent studies report positive prognosis with long-term survival following surgery and chemotherapy, as seen in our patient [7]. The majority of reported cases were recorded over three decades ago and therefore fail to reflect the progress that has been made in diagnostic, imaging, and treatment modalities, which have the potential to facilitate better outcomes within this population. In the 1970s, the cure rate was around 30%, but the development of better neoadjuvant chemotherapy and surgical resection has increased this value to 70% [8]. This highlights a need for further studies that evaluate potential differences in prognostic outcomes and mortality between virilizing and non-virilizing hepatoblastomas.

The surgical management of hepatoblastomas remains complex and subject to ongoing debate. While upfront resection via laparotomy at the time of diagnosis has historically been the standard approach, recent advancements have led to the development of risk stratification systems to guide treatment decisions. Leaders of the four cooperative trial groups (SIOPEL, Children’s Oncology Group, the German Society for Paediatric Oncology and Haematology, and the Japanese Study Group for Paediatric Liver Tumours) developed a risk stratification system that divides patients into four risk groups: very low risk (group A), low risk (group B), intermediate risk (group C), and high risk (group D) [9]. These groups are determined with consideration of the PRETEXT group, a radiologic system that describes the extent of a liver tumor before treatment, age at diagnosis, AFP level, and the presence of a PRETEXT annotation factor [9].

Tumors that are characterized as low risk are resected at the time of diagnosis. However, for tumors that do not fall into this category, the determination of surgery is less straightforward. Surgical intervention is determined after a thorough evaluation of imaging and after neoadjuvant therapy, with general recommendations suggesting that upfront resection be performed only when segmentectomy or nonextended hemihepatectomy with at least 1 cm margins is possible on middle hepatic vein and/or main portal vein division, and there is no concern for macrovascular involvement [9]. However, there is still much debate on whether complete resection is the gold standard, what margins are acceptable, and how/when to manage metastasis.

## Conclusions

The presentation of precocious puberty in hepatoblastoma remains an intriguing paraneoplastic phenomenon, with elevated beta-hCG levels being a key factor in its manifestation. Although virilizing hepatoblastoma is a rare presentation, the growing frequency of reports indicates that there is a need for the incorporation of this presentation as part of the work-up and diagnosis of precocious puberty presentations. There is an increased need to share this presentation with multidisciplinary providers, such as general pediatricians, endocrinologists, and oncologists, to prevent delays in diagnosis, as was seen in this patient, and to treat the underlying cancer.

As surgical management continues to evolve, the adoption of sophisticated risk stratification systems, like those pioneered in the Paediatric Hepatic International Tumour Trial, represents a significant step forward in personalizing treatment strategies. The debate surrounding optimal surgical approaches, whether upfront resection or the timing and extent of surgery following neoadjuvant therapy, remains active. Continued refinement of these protocols, alongside comprehensive studies comparing outcomes across risk categories, will be crucial in enhancing the quality of life for affected patients.
